# Complementary Feeding: Review of Recommendations, Feeding Practices, and Adequacy of Homemade Complementary Food Preparations in Developing Countries – Lessons from Ethiopia

**DOI:** 10.3389/fnut.2016.00041

**Published:** 2016-10-17

**Authors:** Motuma Adimasu Abeshu, Azeb Lelisa, Bekesho Geleta

**Affiliations:** ^1^John Snow, Inc (JSI)-Ethiopia, Addis Ababa, Ethiopia; ^2^Micronutrient Initiative-Ethiopia, Addis Ababa, Ethiopia; ^3^Ethiopian Public Health Institute, Addis Ababa, Ethiopia

**Keywords:** complementary feeding, homemade food, feeding practice, nutrition, breastfed children

## Abstract

Breastfeeding provides the ideal food during the first 6 months of life. Complementary feeding starts when breast milk is no longer sufficient by itself, where the target age is for 6–23 months. The gap between nutritional requirement and amount obtained from breast milk increases with age. For energy, 200, 300, and 550 kcal per day is expected to be covered by complementary foods at 6–8, 9–11, and 12–23 months, respectively. In addition, the complementary foods must provide relatively large proportions of micronutrients such as iron, zinc, phosphorus, magnesium, calcium, and vitamin B6. In several parts of the developing world, complementary feeding continues as a challenge to good nutrition in children. In Ethiopia, only 4.2% of breastfed children of 6–23 months of age have a minimum acceptable diet. The gaps are mostly attributed to either poor dietary quality or poor feeding practices, if not both. Commercial fortified foods are often beyond the reach of the poor. Thus, homemade complementary foods remain commonly used. Even when based on an improved recipe, however, unfortified plant-based complementary foods provide insufficient key micronutrients (especially, iron, zinc, and calcium) during the age of 6–23 months. Thus, this review assessed complementary feeding practice and recommendation and reviewed the level of adequacy of homemade complementary foods.

## Introduction

Exclusive breastfeeding of infants from birth through initial 6 months using breast milk (the ideal food during this period) is important for optimal health, growth, and development (1). As infants grow and become more active following the first 6 months of life, however, breast milk alone falls short of providing the full nutritional requirements – where the gap keeps expanding with the increasing age of the infants and young children (2, 3). Complementary feeding plays critical role in bridging these gaps.

World Health Organization (WHO) defines complementary feeding as “*a process starting when breast milk alone is no longer sufficient to meet the nutritional requirements of infants, and therefore other foods and liquids are needed, along with breast milk*” (4). In order to provide infants with additional nutrients, complementary foods (foods other than breast milk or infant formula) should consequently be introduced to the infants (5).

The target age range for complementary feeding is between the age of 6 and 23 months (with continued breastfeeding), where most infants reach a general and neurological stage of development (chewing, swallowing, digestion, and excretion) that enables them to be fed other foods rather than breast milk (2, 6). Complementary foods could be especially designed transitional foods (to meet particular nutritional or physiological needs of infants) or general family foods, and are expected to address the gaps between the daily energy and nutrient requirement of infants and young children and the amount obtained from breastfeeding (7).

In several parts of the developing world, complementary feeding continues as a challenge to good nutrition in children of 6–23 months (8). In India, for instance, 54.5% of children between the ages of 6 and 8 months had received any complementary foods in the previous day, but only 7% of breastfed children between the ages of 6 and 23 months met the minimum acceptable diet criteria. In Nigeria, only 21% of breastfed children receive the minimum acceptable complementary feeding diet (9). However, in Ethiopia, only 4.2% of breastfed children of 6–23 months of age have a minimum acceptable diet (10). The challenges during complementary feeding are context specific, but many are common across settings. They are often characterized by poor feeding practices and poor dietary quality of homemade complementary foods (11–13).

Poor feeding practices are characterized by poor timing of complementary foods introduction (too early or too late); infrequent feeding; and poor feeding methods, hygiene, and child-care practices (2, 4). Added to these is the poor dietary quality of the foods served, characterized as too little variety; inappropriate consistency (food is too thin or too thick); too few essential vitamins and minerals, especially vitamin A, iron, zinc, and calcium; too few essential fatty acids; and too few calories among non-breastfed infants (12, 14). The poor quality and lack of diversity in foods adversely affects the children’s growth and nutritional status (15).

The objective of this article was to review the energy and nutrient recommendations for infants during complementary feeding period, as well as the feeding practices with primary focus on developing countries that depend on homemade complementary food preparation.

## Overview of Complementary Feeding

During infancy and early childhood (birth to 2 years), adequate amount of appropriate nutrition has paramount importance for full development of children’s human potential. This period is also regarded as “critical window” for child’s health, growth, and development (16). It is also peak period for faltering in child’s growth, micronutrient deficiencies, and emergence of common childhood ailments as diarrhea. Furthermore, reversing of stunting developed during this period is very difficult after the second anniversary of the children (17).

Complementary feeding should be *timely* (start receiving from 6 months onward) and *adequate* (in amounts, frequency, consistency, and using a variety of foods). The foods should be prepared and given in a *safe* manner and be given in a way that is *appropriate* (foods are of appropriate texture for the age of the child) and applying responsive feeding following principles for psychosocial care (2, 6).

During these formative years, poor nutrition has immediate consequences of increased morbidity and mortality and delayed development of the brain and other nervous systems (11). The latent impacts of deficits in nutrients in early ages include impaired cognitive performance and reproductive outcomes and reduced work capacity and health status during adolescence and adulthood. Furthermore, malnutrition cycle persists with intergeneration impacts. When malnourished girl child grows up, she faces greater odds of having malnourished, low birth weight infant (18) – where, the failure to consume additional nutritious food in low resource settings has been identified as important risk factor resulting in excess disease and death of young children (11).

### Age of Introduction of Complementary Foods

It is difficult to pinpoint the ideal time for starting provision to infants of diet other than breast milk (19). There is high risk of harmful effects (possibility of choking, food allergies, and decrease in breast milk intake or formula) through the early introduction of complementary foods. Delayed introduction may miss developmental readiness infants (and difficulties learning to eat at later ages) while risking malnutrition at the same time (5, 20).

According to pediatric nutrition authorities, developmental readiness in most infants and the ability to tolerate foods consumed would occur around 4 and 6 months of age (5, 21). During this period, the intestinal tract will have well-developed defense system that minimizes or averts risk of allergic reaction in infants following intake of foods containing foreign proteins, while its ability to utilize proteins, fats, and carbohydrates improves. Similarly, the infant’s kidney develops to a state where it can successfully eliminate waste products emanating from foods such as meat with characteristic high renal load. Furthermore, their neuromuscular system matures enough leading into development of abilities for recognizing food, accepting spoons, masticating and swallowing foods, and, even, distinguishing and appreciating varieties in food tastes and colors (5, 20, 22).

There is no evidence for harm when safe nutritious complementary foods are introduced after 4 months when the infant is developmentally ready. Similarly, very few studies show significant benefit for delaying complementary foods until 6 months (Table 1) (21).

**Table 1:** **Recommended sequence of introducing complementary foods with food textures and feeding styles by age of infants (5)**.

Age of infant in months	Birth	1	2	3	4	5	6	7	8	9	10	11	12
Age grouping	Birth to 3 months			4–6 months				6–8 months			8–12 months		
Sequence of introducing foods	Breast milk or infant formula			Complementary				Foods					
Texture of complementary foods				Strained/pureed (thin consistency cereal)									
								Mashed					
											Ground/finely chopped		
											Chopped		
Feeding style	Breast feeding/bottle feeding												
				Spoon feeding									
				Cup feeding									
											Self-feeding/feeding finger foods		

Introduction of appropriate diet corresponding to developmental stage of the infants allows provision and intake of sufficient nutrients as per their requirements and facilitates proper development of eating and self-feeding skills. Recommendations for complementary food introduction should follow assessment of infant’s developmental readiness, nutritional status, and health status; the family’s economic and sociocultural issues toward diet and food preferences; and other findings viewed important for consideration (5, 21).

### Consistency of Complementary Foods

The minimum age at which infants develop the ability to swallow particular type of food is highly dependent on their level of neuromuscular development (8). Thus, failure to account such abilities for mastication and swallowing when preparing and serving diets to infants may result in consumption of only trivial amount or extended feeding time (5).

Starting at 6 months, infants can eat pureed, mashed and semi-solid foods prepared from infant cereal, vegetables, fruits, meat, and other protein-rich foods. By 8 months, most infants will become capable of eating “finger foods.” In line with the changing oral skills and emerging new abilities (such as munching, chewing, etc.), the thickness and lumpiness of the foods can gradually change from pureed to ground, fork-mashed, and eventually diced foods (4, 22). Introduction of lumpy solid foods should occur around a critical age window of 10 months so as to avoid latent risk of feeding difficulty associated with late introduction (23).

Evidences suggest that most infants are able to consume solid consistency “family foods” by 12 months, even if they frequently are still served semi-solid foods (24). To enhance optimal growth of the child, it is highly advisable to increase the consistency of the foods gradually with age of the infants even when it would result in longer feeding time for the caregivers. Foods that may cause choking by getting into or blocking airways should be avoided. The risk of chocking from ingesting certain food is often subject to its *size* (small, but hard, pieces that may get into the airway and larger more difficult to chew pieces that may block airways), *shape* (sphere or cylinder shaped that may block airways), and *consistency* (firm, smooth, or slick foods that may slip down the throat; dry or hard foods; sticky or tough foods that may not break apart easily and may be hard to remove from the airways) (4, 5, 7).

## Energy and Nutrient Composition of Complementary Foods

Complementary foods are expected to bridge the gaps in energy and nutrients between daily requirements for infants and young children and the amount consumed through breastfeeding. As such, the diets should be high in energy density, with balanced protein composition (containing all essential amino acids), required vitamins and minerals (iron, folic acid, and calcium), no (safe level) antinutritional components, and while retaining the qualities for palatability (9).

### Energy Requirement

Complementary foods are expected to have sufficient energy density to provide a growing child with adequate daily energy requirement. Energy density is the number of kilocalories of energy in certain food per milliliter per gram of that food. Breast milk contains an energy density of about 0.7 kcal/ml (1).

The recommended minimum energy density in complementary foods is 0.8 kcal/g higher compared to that of breast milk. In reality, the energy density in complementary foods usually is between 0.6 and 1.0 kcal/g and may even drop to as low as 0.3 kcal/g in watery and dilute foods. Consequently, the amount of complementary food required to cover the energy gap corresponds to the level of energy density in the diets served (7).

Energy-dense foods are most important for children with wasting, as they have an increased energy need for catch-up growth. Low energy density complementary foods have long been implicated in PE malnutrition (25).

**Figure 1:**
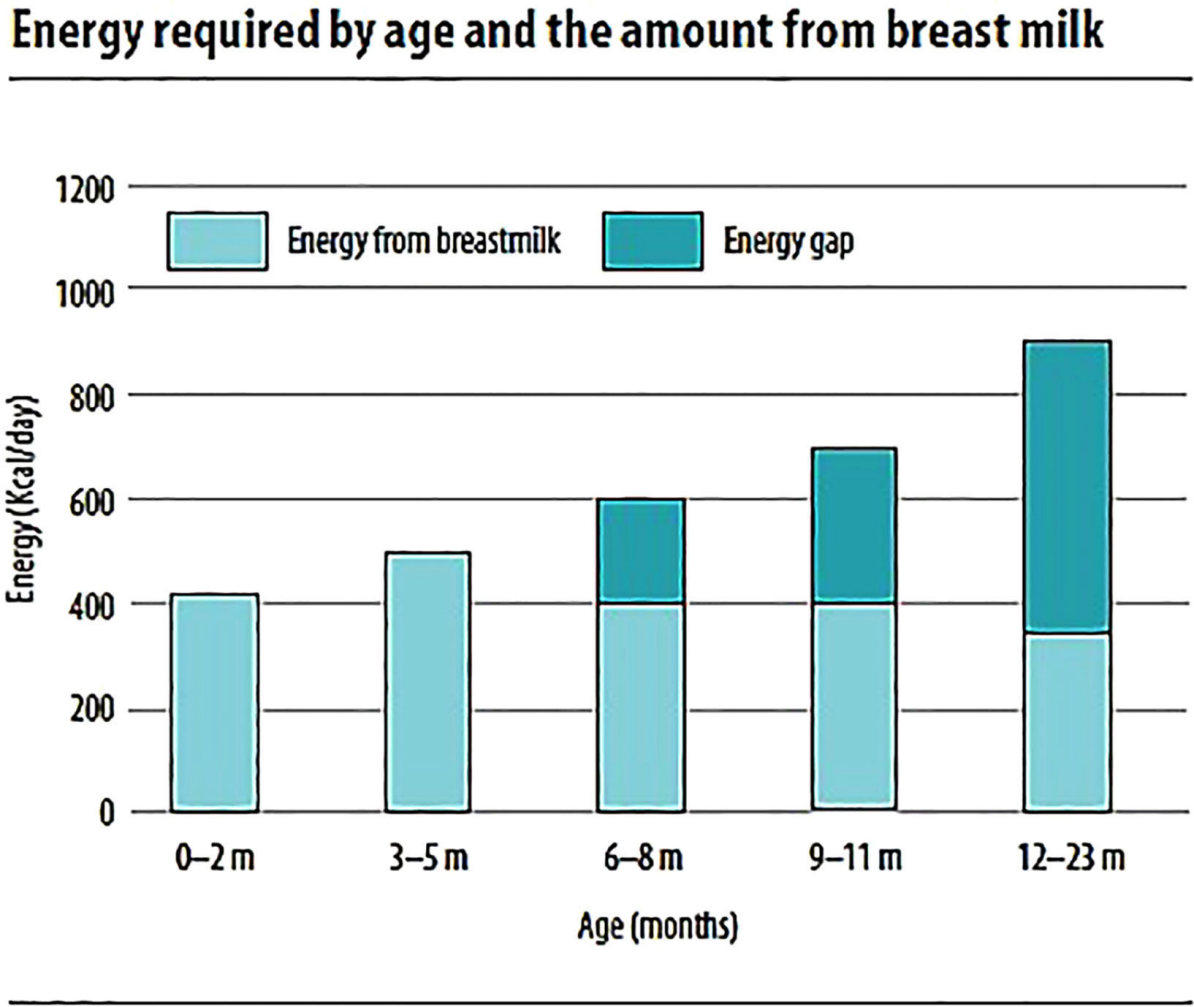
**Energy required by age and the amount from breast milk (7)**.

The total energy requirement estimated for healthy breastfed infants is approximately 615 kcal/day at 6–8 months, 686 kcal/day at 9–11 months, and 894 kcal/day at 12–23 months (3). For infants in developing countries with “average” breast milk intake, the energy need from complementary food increases from 200 kcal/day at 6–8 months to 300 and 500 kcal/day at 9–11 and 12–23 months, respectively (Figure 1). This accounts for 29, 55, and 71% of total daily energy needs, respectively, coinciding with the decreased intake of human milk at older ages. These values could vary based on the level of breast milk intake per day (3, 4).

The amount of food needed per day to satisfy their energy requirement is a function of amount of energy needed from complementary foods and the energy density of the foods (i.e., kilocalories per gram) (7, 26). For complementary foods with energy density range of 0.6–1 kcal/g, the amount (gram or volume) of food needed to provide energy requirement is 200–333 g/day for 6- to 8-month-old, 300–500 g/day for 9- to 11-month-old, and 550–917 g/day for 12- to 23-month-old children. Energy-dense foods have energy density of 1.07–1.46 kcal/g. For such foods, the approximate quantity of complementary food that would meet the energy needs described above is 137–187 g/day for 6–8, 206–281 g/day for 9–11, and 378–515 g/day for 12- to 23-month-old children (4).

The number of meals per day is dependent on the energy gap for stated age, gastric capacity of the child, and energy density of the meal (kilocalories per gram). Thus, for a given age interval and level of breast-milk intake, calculating the recommended number of meals requires information about the energy density of the foods. For older children requiring larger quantity of food in a day, the food needs to be sub-divided multiple servings compared to their younger counterparts (7).

For foods containing recommended minimum energy density (0.8 kcal/g), assuming gastric capacity of 30 g/kg body weight, the meal frequency expected to provide adequate daily energy requirement is two to three times for 6–8, and three to four times for 9–11 and 12- to 24-month-old children, with one to two nutritious snacks (4). Transition from two to three meals or smaller to larger meals between the ages should happen gradually based on appetite and development of the child (7).

### Protein Requirement

Protein makes important nutrient composition in complementary foods. They are major sources of essential amino acids and energy at times of energy deprivation. Adequate supply of dietary protein is vital for maintaining cellular function and integrity and for ensuring normalcy of health and growth. On the other hand, the combined effect of protein deficiency and low energy intake leads to protein–energy (PE) malnutrition, the commonest forms of malnutrition worldwide (27).

The protein requirement of infants and young children increases with their age. The amount of protein (in grams per day) required to satisfy their daily nutritional requirement is 9.1 g for 6–8 months, 9.6 g for 9–11 months, and 10.9 g for 12–23 months. Breast milk provides a significant portion of daily protein requirement of infants and young children. When average breast-milk intake is assumed, the amount of protein needed from complementary foods is 1.9 g/day at 6–8 months (21%), 4.0 g/day at 9–11 months (42%), and 6.2 g/day (57%) at 12–23 months (3, 4, 8).

The PE ratio, which is the ratio of energy from protein to total energy contained in certain amount of food, is one of the indicators used to assess the quality and adequacy of protein in complementary foods. Based on the protein quality of the food (high quality, such as milk, or lower quality, such as plants) and age of children, the recommendations for PE ratio vary. Expressed as a percentage of estimated energy requirements coming from protein, some countries set a PE ratio range of 8–15% energy from protein (i.e., 2–3.75 g protein per 100 total kilocalories). The PE ratio is approximately 7.5% in human milk and 8–8.5% in infant formulas. These ratios are adequate for a normal rate of growth (24, 28).

The minimum recommended PE ratio to come from complementary foods of children between 6 and 23 months of age lies between 4.3% (for foods with high protein quality, such as milk, and for child of age 23 months) and 6.3% (for foods with low protein quality, such as plants, and for a child of age 6 months) (26, 28).

### Fats/Lipids Requirement

Dietary fats constitute an important portion of nutrients obtained from foods. For infants and young children, they are source of energy, essential fatty acids, and fat soluble vitamins (A, D, E, and K). In addition, dietary fats have an important role in promoting good health and enhancing the sensory qualities of the foods (27).

Fat accounts for about 50% of breast-milk’s energy and serves as primary energy source for infants during the first 6 months of life. With the introduction of complementary food, however, fat is gradually overtaken by carbohydrate as the chief energy source. Even so, fat remains important source of energy, and together with carbohydrates, they meet the energy needs of the growing child (4, 6).

There is often a debate on the optimal amount of fat in the diets of infants and young children. Although limited evidences exist, average daily fat equivalent to 30–45% of energy intake is often suggested to balance the compromise in risks from little intake (especially essential fatty acids and lowered energy density levels), and excess intake and the likelihood of childhood obesity and cardiovascular diseases (4, 29). This recommendation also guarantees adequate intake of essential fatty acids, fat soluble vitamins, and improved energy (4, 7).

If the percentage of energy from fat is accounted to be at least 30%, the amount of fat needed from complementary foods to satisfy daily requirement for infants and young children depends on the level of breast milk intake. For those with low breast milk intake, complementary foods should provide dietary fats appropriating to 34, 38, and 42% of daily energy requirements for 6–8, 9–11, and 12–23 months, respectively. With adequate breast milk intake, however, the requirement from complementary foods is 0 g/day (0%) at 6–8 months, 3 g/day (5–8%) at 9–11 months, and 9–13 g/day (15–20%) at 12–23 months (4).

### Carbohydrates

Starch is likely to be a major constituent of many complimentary foods for older infants and young children. To ensure that its energy value is realized, this starch should be provided in a readily digestible form. Increasing the intake of dietary fibers increases stool bulk, causes flatulence, and decreases appetite. Lack of agreement on the definition of fiber and differences in analytical techniques make it difficult to compare recommendations from different sources. Infants consume a very low-fiber diet, although oligosaccharides in breast milk are thought to have fiber-like properties. Fibers should be introduced gradually into their diet from the age of 6 months. Use of large quantities of whole grain cereals and pulses or nuts during infancy is not recommended as they are likely to affect bioavailability of micronutrients and result in a low-energy diet (26).

The extent to which foods produce satiation and sustain satiety depends, in part, on their nutrient composition. Proteins and lower energy density foods are considered much satiating. Similarly, high-fiber foods effectively provide satiation by filling the stomach and delaying the absorption of nutrients. Such attributes of the complementary foods may also lower the child’s feeding ability (27).

### Micronutrients

Micronutrients are essential for growth, development, and prevention of illness in young children (7). Adequate intakes of micronutrients, such as iron, zinc, and calcium, are important for ensuring optimal health, growth, and development of infants and young children (26, 27).

**Figure 2:**
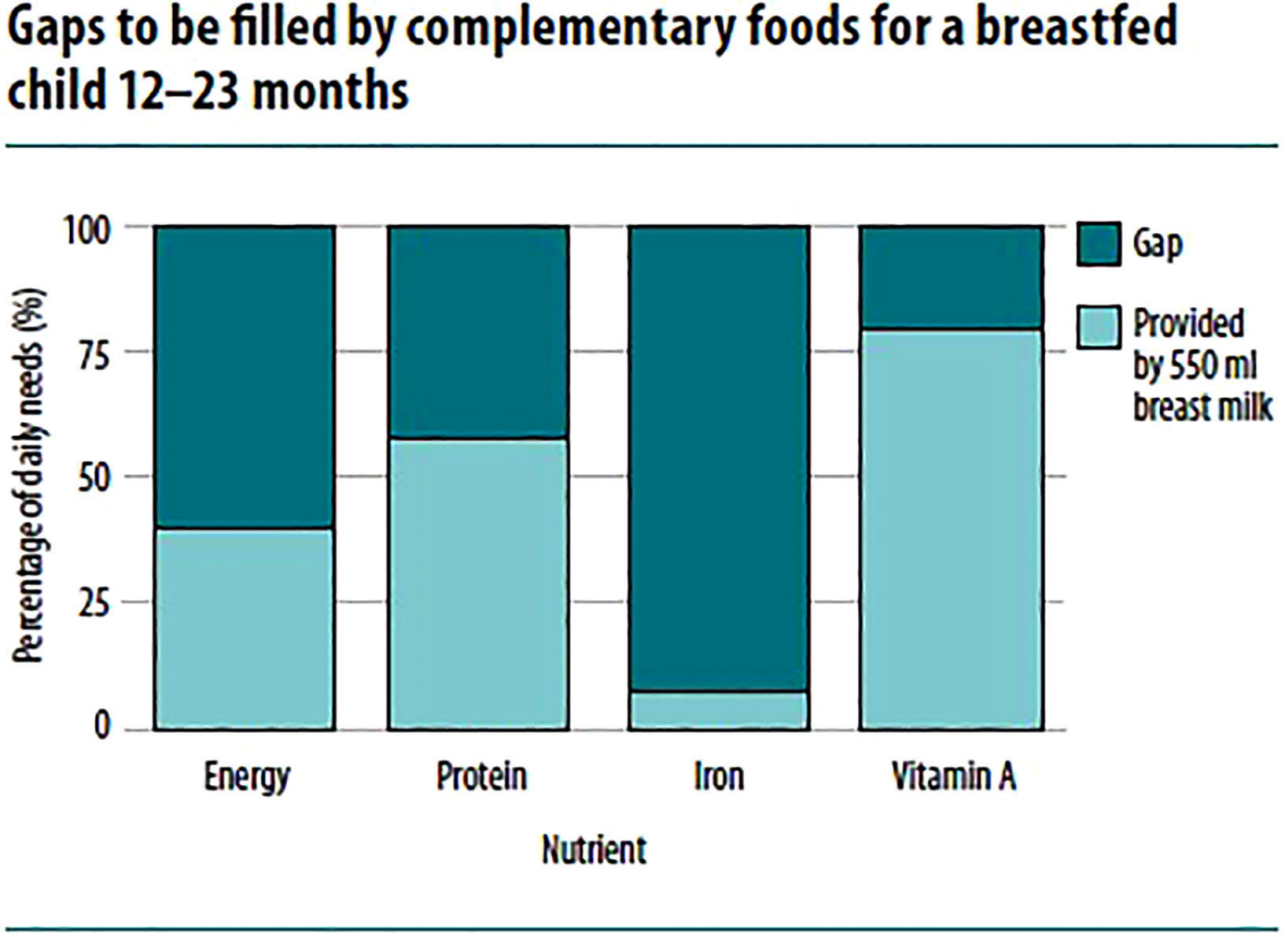
**Gaps to be filled by complementary foods for a breastfed child 12–23 months (7)**.

Breast milk makes substantial contribution to the total nutrient intakes. In well-nourished mothers, breast milk contains generous amounts vitamin A, B, C, folate, iodine, and selenium. As a result, the amount needed from complementary foods before 12 months is 0 (or close to 0) (Figure 2) (3, 29).

However, breast milk is relatively low in several other micronutrients, even after accounting for bioavailability. The percentage of total daily requirement for micronutrients needed from complementary foods ranges from 30 to 97%. For instance, 97% of iron, 86% of zinc, 81% of phosphorus, 76% of magnesium, 73% of sodium, and 72% of calcium during 9–11 months are expected from complementary foods (3, 4, 29). Thus, added to the fact that infants bear only limited gastric capacity to consume adequate quantity of food, the diets need to have very high nutrient density (4).

## Food Items Used to Prepare Complementary Foods

From the sixth month onward, complementary foods should be of variety, and balanced mixtures of food items containing cereals, tubers, foods of animal and vegetable origin, and fat should be offered. Only a varied diet guarantees the supply of micronutrients, enhances good eating habits, and prevents the development of anorexia caused by monotonous foods (6, 7, 30).

Grain products (whole grain) can serve as sources for carbohydrates, fibers, and micronutrients such as thiamin, niacin, riboflavin, and iron. Protein-rich foods, such as meat, poultry, fish, egg yolks, cheese, yogurt, and legumes, can be introduced to infants between 6 and 8 months of age. Fruits and vegetables introduced over time can provide infants with carbohydrates, including fiber, vitamins A and C, and minerals (6, 23, 30).

Complementary foods usually are of two types: commercially prepared infant foods bought from the market and homemade complementary foods, which are prepared at household level by the caregivers following traditional methods.

Commercially, complementary foods can be produced following simple technologies such as malting, popping, fermentation, or using modern food-processing technologies such as roller drying and extrusion cooking. Some of the commonly available commercially prepared infant foods include iron-fortified infant cereal made of food items, such as *rice*, oat, and barley, wheat, mixed-grain infant cereals, and infant cereal and fruit combinations; juices such as infant juices, citrus juices, canned juices, and unpasteurized juices; commercially prepared vegetable or fruit infant foods; and commercially prepared infant food meats (31, 32).

## Homemade Complementary Foods

Complementary foods could also be prepared at household level by the caregivers following other traditional methods. These are commonly described as homemade complementary foods. The recommendation for specific food type to prepare depends on their age appropriateness and development stage of infants and young children.

For infants and young children of age 6–11 months, for instance, provision of *thick porridge* made of maize, cassava, millet; to which milk, soy, ground nuts, or sugar is added; or *mixtures of pureed foods* made out of potatoes, cassava, *posho* (maize or millet), or rice, being mixed with fish, beans or pounded groundnuts, and green vegetables added would allow consumption of nutritious foods. Addition of nutritious snacks, such as egg, banana, bread, papaya, avocado, mango, other fruits, yogurt, milk, and puddings made with milk, biscuits or crackers, bread or *chapatti* with butter, margarine, groundnut paste or honey, bean cakes, and cooked potatoes, would suffice their nutritional needs (7, 8, 16, 30, 33).

For children of 12–23 months, provision of adequate servings of *mixtures of mashed or finely cut* family foods made out of *matoke*, potatoes, cassava, posho (maize or millet), or rice; mix with fish or beans or pounded groundnuts; add green vegetables or *thick porridge* made out of maize, cassava, and millet; add milk, soy, ground nuts, or sugar, would allow consumption of nutritious foods. Addition of nutritious snacks, such as egg, banana, bread, papaya, avocado, mango, other fruits, yogurt, milk, and puddings made with milk, biscuits or crackers, bread or *chapatti* with butter, margarine, groundnut paste or honey, bean cakes, and cooked potatoes, would suffice their nutritional needs (Table 2) (7, 8, 30, 33).

**Table 2:** **Practical guidance on the quality, frequency, and amount of food to offer children 6–23 months of age (7)**.

Age	Energy needed per day in addition to breast milk	Texture	Frequency	Amount of food an average child will usually eat at each meal
6–8 months	200 kcal per day	Start with thick porridge, well-mashed foods	2–3 meals per day	Start with 2–3 tablespoonful per feed, increasing gradually to 1/2 of 250-ml cup
		Continue with mashed family foods	Depending on the child’s appetite, 1–2 snacks may be offered	
9–11 months	300 kcal per day	Finely chopped or mashed foods and foods that baby can pick up	3–4 meals per day depending on the child’s appetite, 1–2 snacks may be offered	1/2 of a 250-ml cup/bowl
			Depending on the child’s appetite, 1–2 snacks may be offered	
12–23 months	550 kcal per day	Family foods, chopped or mashed if necessary	3–4 meals per day	1/4 to full 250-ml cup/bowl
			Depending on the child’s appetite, 1–2 snacks may be offered	

The complementary foods should also contain foods rich in *iron*: liver (any type), organ meat, flesh of animals (especially red meat), flesh of birds (especially dark meat), and foods fortified with iron; *vitamin A, K*: liver (any type), red palm oil, egg yolk, orange colored fruits and vegetables, and dark green vegetables; *zinc*: liver (any type), organ meat, food prepared with blood, flesh of animals, birds, and fish, shell fish, and egg yolk; *calcium*: milk or milk products and small fish with bones; and *vitamin C*: fresh fruits, tomatoes, peppers (green, red, and yellow), and green leaves and vegetables (7, 8, 16, 30, 33).

In many developing countries, commercial fortified food products are often beyond the reach of the poor. As a result, homemade complementary foods are frequently used during child feeding (34). The basic recipe food items used for the preparation of the complementary food commonly base on locally available staples, while the choice of specific food item differs considerably between populations, owing to tradition, availability, and ease of access (35). In many developing countries, the staples are cereals, roots, and starchy fruits that consist mainly of carbohydrate and provide energy (34).

Cereals form the staple foods of virtually all populations. Cereals are an important source of energy, providing between 334.4 and 382.2 kcal/100 g of whole cereal, and provide starch and dietary fibers (soluble and insoluble). Grains comprise 70–77% of all cereals, which usually are processed and cooked to make the starch more digestible (26).

In cereals, 65–75% of the total weight is carbohydrate, 6–12% is protein, and 1–5% is fat. The protein quality, however, is very low compared to animal-based foods (31). For instance, the protein composition of maize and guinea corn used in several West African countries is of poor protein quality and low in lysine and tryptophan. In Nigeria, corn gruel contained only 0.5% protein and less than 1% fat, compared to 9% protein and 4% fat in the original corn, and has been indicated to have been too low even to support the growth of rat (36, 37).

In several West African countries, the first solid food and popular complementary foods are based on thin cereal and are low in foods from meat, eggs, or fish, especially among low-income groups due to socioeconomic factors, taboos, and ignorance (37).

In Nigeria, for instance, such foods are made from maize (*Zea mays*), millet (*Pennisetum americanum*), or guinea corn (*Sorghum* spp.). After successful introduction of cereal gruel, other staple foods in the family menu, such as yam (*Dioscorea* spp.), rice (*Oryza sativa*), *gari* (fermented cassava grits), and cocoyam (*Xanthosoma sagittifolium*), are given to the child being mashed, thinned, or pre-chewed. Legumes are rarely used and are introduced much later (after 6 months of age) because of the problems of indigestibility, flatulence, and diarrhea associated with their use (36, 37).

In Ghana, the main complementary food for infants up to 6 months of age was a traditional fermented maize porridge (*koko*). From 6 months onward, the infants were given the family diet with supplementary breastfeeding. The family foods, including dishes made from cereal, starchy tubers, legumes, and vegetables, were used when introducing complementary foods (37).

In Guatemala, such family foods used as complementary foods are significantly short in micronutrients, such as iron, zinc, and calcium, even if adequate amounts of protein, B vitamins [vitamins B-1 (thiamine), B-2 (riboflavin), B-6, and B-12], and vitamin C would be supplied (38).

A commonly shared phenomenon about homemade complementary foods that are based on starchy roots and tubers or rice and available in many low-income countries is their frequent shortfall in amounts of selected essential micronutrients such as calcium, iron, and zinc. In contrast, the recipes prepared from maize and legumes or other cereal mixtures and legumes had higher iron and zinc contents, but they also have considerably higher phytate contents (39). Under both circumstances, they fail to meet the theoretical mineral requirements of young children due either to their low mineral content or as a result of low bioavailability, unless enriched with animal-source foods such. As a result, WHO designates calcium, iron, and zinc as “problem nutrients,” and deficiencies of these minerals can lead to adverse health consequences and restricted child growth and development (4, 39).

Complementary foods need to be far more nutrient-rich compared to family foods. Yet, the opposite is the case in low-income households. The foods are often known to be of low nutritive value and are characterized by low protein, low energy density, and high bulk (40). Bulk is one of the major problems of homemade complementary foods, where a problem of high viscosity, low energy density, or both may occur (4). Under such circumstances, it is usually possible to achieve an adequate protein and energy intake for adults and older children by increasing the daily intake. For infants and younger children, however, the volume of the diets may be too large to allow the child to ingest all the food necessary to cover his or her energy needs. For instance, a 4- to 6-month infant would need 62 g of corn gruel to meet daily needs of energy (740 kcal) and protein (13 g), which would be an impossible task considering the size of an infant’s stomach (37, 41).

South African mothers often use soft, bulky, and low nutrient density maize meal porridge to introduce solids to their infants. The maize meal is typically diluted to thin consistency, using water, which further lowers the nutrient density and increases the bulk of the food. Being high in its phytate composition, the absorption and bioavailability of iron and zinc is greatly hampered (13). Similarly, in Philippines, complementary foods are prepared as thin porridges, thus having low energy and nutrient density. As a result, the median intakes (per day; per 100 kcal) of energy, calcium, iron, zinc, and vitamins A and C (but not protein) by infant and young children failed to meet WHO estimated needs and desired nutrient densities (42).

### Homemade Complementary Foods in Ethiopia

The recommendation for complementary feeding recipes for Ethiopian children of 6–23 months are based on simple and locally available food items, but not that nutritious enough to fill the calorie, protein, and micronutrient gap between total daily needs and the amount provided by the breast milk. The recipes are based on three major staples that are locally available, including maize/*enset*/*teff*, wheat/barley, and sorghum/maize (33), while the target energy and nutrient composition and daily intakes from the complementary foods were drawn in line with the WHO’s infant and young child feeding/complementary feeding recommendations (3, 4, 8, 33).

Homemade complementary foods in Ethiopia predominantly are based on cereals and legumes and mostly an extension of family foods. The complementary foods are made from staple cereals or starchy tubers such as maize, sorghum, millet, oat, *teff*, rice and yam, potato, and barley and yam. The usual complementary foods are served as gruel, porridge, *fetfet, kitta, and dabo*. Consumption of animal-source foods as well as fruits and vegetables is very low (Table 3) (41, 43).

**Table 3:** **Major grain-based traditional complementary foods (41)**.

Complementary foods	Raw food items used
Gruel	Teff, sorghum, barley, maize, wheat, emmer wheat, and enset
Porridge	Teff, sorghum, barley, maize, wheat, emmer wheat, and enset
Fetfet	Teff, sorghum, barley, maize, wheat, broad beans, chick peas, field peas, and lentil
Kitta	Teff, sorghum, barley, maize, wheat, enset, and chick peas
Dabo	Teff, sorghum, barley, maize, wheat, and emmer wheat

Literatures suggest that unfortified complementary foods that are predominantly plant-based generally provide insufficient amounts of certain key nutrients (particularly iron, zinc, and calcium) to meet the recommended nutrient intakes during 6–23 months of age (8, 29, 44).

Animal-source foods are rich in protein, fat, and micronutrients, and their inclusion can therefore meet the gap in some cases. However, this might be impractical for lower income groups, given their high cost. Even when availed, the amount of food from animal sources that children can consume at ages of 6–12 months would become insufficient to facilitate intake of adequate nutrients as iron, calcium, and, seldom, zinc – in comparison to the nutrient gaps (7). Gibson et al. (44) evaluated 23 different complementary food mixtures used in developing countries, including the ones consumed in Ethiopia, some of which included animal-source foods. None of the complementary foods achieved the desired nutrient density for iron, and while few achieved the desired level for calcium or zinc.

As has been observed in most other developing countries, the traditional complementary foods have been reported to be grossly inadequate when compared to estimated needs. Existing literature for the nutrient density, energy levels, and adequacy of the amount consumed in comparison to their nutritional requirement are not consistent (41, 43–45).

Gibson et al. (44) conducted analysis of nutrient density and energy composition of complementary food preparation, for children between 9 and 11 months, from starchy roots, tubers, and similar foods. The foods were prepared in a laboratory using recipe collected from the field, including such food items as potato, kale, chickpea flour, oil, and water, with formulation ratio of 63:17:8:2:10. The nutrient composition and adequacy was then analyzed based on an estimated food record method. Findings of the assay show that the complementary foods have protein density of 2.3 g/100 kcal and energy density of 123 kcal/100 g as eaten (on fresh basis). Assuming meal frequency of three times day, and having individual servings of at least 250 ml, intake for energy and protein meets the estimated need to fulfill the requirement of infant and young child between 9 and 11 months.

On the other hand, Abebe et al. (45) reported that traditional corn-based porridge consumed as complementary foods in rural villages of Sidama zone of Southern Ethiopia contain energy density of 53 kcal/100 g, while kocho-based ones contained 49 kcal/100 g, both of which containing very low energy levels. With this energy density, however, the children would require nearly twice the amount of food intakes compared to the amount needed from foods containing energy density of 0.8–1.0 kcal/g (4, 45).

In another study, nutrient intakes from complementary foods among children of age 12–23 months in North Wollo, Northern Ethiopia, was found to be short of the requirement. When the amount of nutrients consumed from complementary foods per day was compared with their daily requirement, intake of energy was found to be lower than estimated needs. The nutrient density for protein, however, met the desired level (43).

Iron, zinc, and calcium are limiting nutrients in unfortified plant-based complementary foods commonly used in developing countries (8, 29, 44). According to Baye et al. (43), calcium in the complementary foods collected from North Wollo was below the desired values. However, the nutrient density for iron meets the desired values even under the assumption of low bioavailability, while zinc meets the nutrient density requirement when accounting for a moderate bioavailability. While the children consumed sufficient amount of protein and iron, they fell short of their calcium and zinc needs from complementary foods.

In contrast to this, Gibson et al. (44) reported that the complementary foods under study failed to meet the desirable level for Ca, Zn, and Fe, even if moderate bioavailability is assumed for iron and zinc, and would not satisfy the estimated daily requirement for these nutrients for children of 9–11 months, even if ideal serving sizes are provided.

## Conclusion

Homemade complementary foods in Ethiopia are based predominantly on cereals and legumes, and are mostly an extension of family foods, such as *fetfet, kitta, dabo*, gruel, and porridge. As such, the diets have good energy density even if adequacy of daily energy intake was conflicting. However, the diets are very low in nutrient density of micronutrients, including those labeled by the WHO as “problem nutrients.” The diets lack animal-source foods as well as fruits and vegetables. Intake of micronutrients, such as iron, zinc, and calcium, from homemade complementary foods was very low.

## Limitations

This paper tried to review recommendations and practices revolving around complementary feeding. Even if many important areas were covered, the following limitations should also be noted. First, the review focuses on recommendations for full-term children with average breast milk intake and does not address requirement for non-breastfed and non-full-term children. In addition, due emphasis was given to feeding practices based on homemade complementary foods in developing countries such as Ethiopia.

## Author Contributions

MAA reviewed literature and wrote the manuscript. AL and BG reviewed the manuscript. All authors approved the final manuscript.

## Conflict of Interest Statement

The authors declare that the research was conducted in the absence of any commercial or financial relationships that could be construed as a potential conflict of interest.
